# Vancomycin and Clarithromycin Show Synergy against *Mycobacterium abscessus In Vitro*

**DOI:** 10.1128/AAC.01298-17

**Published:** 2017-11-22

**Authors:** Devika Mukherjee, Mu-Lu Wu, Jeanette W. P. Teo, Thomas Dick

**Affiliations:** a Department of Microbiology and Immunology, Yong Loo Lin School of Medicine, National University of Singapore, Singapore; b Department of Medicine, Yong Loo Lin School of Medicine, National University of Singapore, Singapore; c Department of Laboratory Medicine, National University Hospital, Singapore; d Public Health Research Institute, New Jersey Medical School, Rutgers, The State University of New Jersey, Newark, New Jersey, USA

**Keywords:** Mycobacterium abscessus, clarithromycin, synergy, vancomycin

## Abstract

Lung disease caused by Mycobacterium abscessus is increasing, and current clarithromycin-based treatment regimens are only moderately effective. Here, we determined the effect of clarithromycin-vancomycin combination against M. abscessus complex isolates *in vitro*. Synergy was found with a fractional inhibitory concentration index (FICI) score of ≤0.5 and a 4- to 10-fold decrease in MIC.

## TEXT

The rapidly growing Mycobacterium abscessus gained importance as a pathogen in the early 1990s (1). M. abscessus mostly causes lung infections in vulnerable populations, including patients with cystic fibrosis, but also causes skin, soft tissue, and ocular infections (2). Patterns of drug resistance differ among the three currently recognized subspecies of the complex M. abscessus subsp. abscessus, M. abscessus subsp. bolletii, and M. abscessus subsp. massiliense (3, 4). Hence distinguishing between the subspecies is important clinically because they respond differently to antibiotic therapy (5, 6). Treatment regimens are combination based and rely on a macrolide (usually clarithromycin) and an aminoglycoside (such as amikacin) along with another active antimicrobial, such as a β-lactam (imipenem, cefoxitin) (7). Therapy is lengthy, requiring negative sputum cultures for 1 year (8), and often unsuccessful. Treatment failure is often attributed to clarithromycin resistance that is prevalent in the M. abscessus complex (1). Constitutive resistance to macrolides occurs due to point mutations at position 2058 or 2059 of the 23S rRNA (*rrl*) gene (9, 10). Inducible macrolide resistance in M. abscessus subsp. abscessus and M. abscessus subsp. bolletii is conferred by the ribosomal methylase gene *erm*(41) (11). However, the functionality of the *erm*(41) gene in these two subspecies is complicated by sequevars that carry a T→C substitution at position 28 of its coding sequence. A C28 *erm*(41) sequevar is nonfunctional and hence susceptible to clarithromycin (11). On the other hand, M. abscessus subsp. massiliense usually possesses a truncated, nonfunctional *erm*(41) gene copy and thus does not display inducible macrolide resistance (9, 11).

Previously, we screened a library of U.S. Food and Drug Administration-approved drugs for activity against M. abscessus and found that the glycopeptide vancomycin showed weak growth inhibitory activity against the clinical isolate M. abscessus Bamboo (12). Vancomycin is a tricyclic glycopeptide antibiotic from Amycolatopsis orientalis and is commonly used against Gram-positive bacteria. It prevents the cross-linking of *N*-acetylglucosamine/*N*-acetylmuramic acid peptides, thus inhibiting peptidoglycan synthesis of the cell wall (13). This results in bacterial cell wall weakening and thus may facilitate increased penetration of other antimicrobials and enhance their potency. Here, we asked whether vancomycin can be used to enhance clarithromycin potency and examined the *in vitro* synergistic potential of these two drugs against M. abscessus complex type strains and clinical isolates.

Type strains M. abscessus subsp. abscessus ATCC 19977, M. abscessus subsp. bolletii Culture Collection University of Gothenburg (CCUG) 50184T, and M. abscessus subsp. massiliense CCUG 48898T (*n* = 3) and clinical isolates (*n* = 9) were used for susceptibility and synergy testing (Table 1). Cultures were grown as described previously (12). Whole-genome sequencing of M. abscessus Bamboo showed that this strain represents a C28 sequevar, rendering the isolate sensitive to clarithromycin (14). The other eight clinical isolates were obtained from the strain collection of the clinical microbiology laboratory at the National University Hospital, Singapore. The isolates were speciated using *rpoB* and *hsp65* (15), as described previously (12). The *erm*(41) and *rrl* genes were analyzed as previously described (12). The 28th nucleotide of *erm*(41) was examined for T/C polymorphisms. The presence of mutations at nucleotide positions 2058 to 2059 of *rrl* encoding the 23S rRNA were also examined. Mutations at these positions are responsible for constitutive clarithromycin resistance. None of the isolates used in this study possessed *rrl* mutations.

**TABLE 1:** *In vitro* synergy of vancomycin and clarithromycin in M. abscessus complex isolates

Isolate code	M. abscessus subspecies^*a*^	*erm*(41) sequevar^*a*^	MIC (μM) of^*b*^:				FICI
			CLR alone	VAN alone	CLR in combination	VAN in combination	
ATCC 19977	abscessus	T28	6.25	25	0.39	3.12	0.19
CCUG 50184T	bolletii	T28	12.5	12.5	0.78	1.56	0.19
CCUG 48898T	massiliense	Deleted	1.56	50	0.39	3.12	0.31
Bamboo	abscessus	C28	1.56	25	0.20	3.12	0.25
M337	abscessus	T28	6.25	100	0.78	12.5	0.25
M199	abscessus	T28	12.5	100	1.56	12.5	0.24
M404	abscessus	C28	0.78	6.25	0.20	1.56	0.49
M9	abscessus	T28	6.25	100	1.56	6.25	0.31
M422	abscessus	T28	3.12	25	0.39	6.25	0.37
M111	massiliense	Deleted	0.39	6.25	0.10	1.56	0.49
M506	bolletii	C28	1.56	200	0.39	50	0.50
M232	bolletii	T28	12.5	50	3.12	12.5	0.49

a Subspecies were determined by sequencing *rpoB* and *hsp65*; *erm*(41) sequevar was determined by sequencing the gene.

b Experiments were repeated twice independently, and mean values are shown. Standard deviations were ±50% of the shown values. VAN, vancomycin; CLR, clarithromycin.

Vancomycin was dissolved in water and clarithromycin in acetone. The effect of acetone on growth of the bacteria was tested up to a concentration of 2% (equivalent to the highest concentration used) and was found to have no effect on the growth of the bacteria. MICs were determined using 2-fold serial dilutions of the drugs in 96-well microtiter plates that were incubated with the bacterial cultures at 37°C for 72 h. After this, optical density at 600 nm (OD_600_) was measured using the Tecan infinite Pro 200 plate reader. OD_600_ was used as a measure of growth, and growth inhibition was assessed in comparison to growth in drug-free wells. The concentration of each drug that inhibited 90% growth was defined as its MIC. The interaction between clarithromycin and vancomycin was assessed using a broth microdilution checkerboard assay. The concentrations of the drugs used were decided based on MICs and were within the range of 0.05 to 200 μM for both vancomycin and clarithromycin. Fractional inhibitory concentration index (FICI) was calculated as (MIC of drug A in combination/MIC of drug A alone) + (MIC of drug B in combination/MIC of drug B alone), where A is clarithromycin and B is vancomycin. Synergy was defined as a FICI score of ≤0.5 (16).

The MICs of the drugs alone and in combination are presented in Table 1. The MIC of vancomycin ranged from 6.25 to 200 μM, and that of clarithromycin ranged from 0.39 to 12.5 μM. The MICs of both drugs were reduced by 4- to 10-fold when tested in combination. Synergy was demonstrated for all of the laboratory strains and the clinical isolates. Synergy was also evaluated on induction of clarithromycin resistance, which was achieved by preincubating bacterial cultures with clarithromycin for 24 h at 0.1 μM (a concentration that does not inhibit bacterial growth on its own) as previously described (12). This preincubation assay resulted in a shift in the clarithromycin MIC to >200 μM. The two subspecies type strains with inducible clarithromycin resistance (ATCC 19977 and CCUG 50184T) were tested after preincubation with clarithromycin, and both showed a dramatic reduction in clarithromycin MIC, from >200 to 0.39 μM for M. abscessus subsp. abscessus and 3.12 μM for M. abscessus subsp. bolletii, when combined with vancomycin (Table 2). When combined with clarithromycin, the vancomycin concentration was 4-fold lower than with vancomycin alone. In addition, the standard inducible resistance assay for clarithromycin with prolonged incubation was carried out as described by Nash et al. (11) (Table 2). Similar to the results with the preincubation assay, at 14 days, the MIC for clarithromycin alone was >200 μM. In combination with vancomycin, clarithromycin MIC shifted to 12.5 and 25 μM for M. abscessus subsp. abscessus and M. abscessus subsp. bolletii, respectively.

**TABLE 2:** *In vitro* synergistic effect of clarithromycin and vancomycin against two M. abscessus type strains with inducible clarithromycin resistance under induced conditions

Strain	Day	MIC (μM) of:				FICI^*a*^
		CLR alone	VAN alone	CLR in combination	VAN in combination	
Preincubation induction assay						
M. abscessus subsp. abscessus ATCC 19977	3	>200	12.5	0.39	3.12	<0.25
M. abscessus subsp. bolletii CCUG 50184T	3	>200	6.25	3.12	1.56	<0.25
Standard inducible resistance assay						
M. abscessus subsp. abscessus ATCC 19977	3	6.25	25	0.39	3.12	0.19
	7	25	100	0.39	12.5	0.14
	14	>200	100	12.5	6.25	<0.13
M. abscessus subsp. bolletii CCUG 50184T	3	12.5	12.5	0.78	1.56	0.19
	7	25	50	1.56	3.12	0.12
	14	>200	50	25	1.56	<0.16

a A less than symbol (<) preceding a FICI score indicates that an MIC of the drug alone was higher than the greatest concentration tested, which was used in FICI calculation.

Taken together, our results indicate that combinations of vancomycin and clarithromycin *in vitro* have synergistic effects and may thus be useful in treating M. abscessus infections. To our knowledge, this is the first report demonstrating vancomycin-clarithromycin synergy in M. abscessus. The reduced MICs may facilitate achievement of clinically useful *in vivo* drug concentrations and thus make therapy more effective. The observed synergy may also allow for lower doses of either antibiotic and hence reduce toxicity (17).

Patients infected with strains possessing an intact *erm*(41) gene have a lower rate of successful treatment outcomes (18, 19). Here, using our preincubation assay and the standard inducible resistance assay with prolonged incubation, we were able to demonstrate synergy with this combination in the presence of the intact *erm*(41) gene. These results suggest that the clarithromycin-vancomycin combination may be useful in improving the treatment outcomes of infections involving organisms that harbor inducible clarithromycin resistance.

A limitation of using this combination is that there are no established breakpoints for M. abscessus infection. Another factor to consider is that vancomycin is administered intravenously (20), which makes adherence an issue due to the long treatment duration of M. abscessus infections. Whether this combination can be applied clinically is debatable, as it remains to be determined whether vancomycin MICs in combination are achievable in human serum and whether the toxicity will be reduced by the use of combination therapy. However, the synergistic effect seen between vancomycin and clarithromycin establishes the grounds for further investigation into the use of vancomycin-like antibiotics, such as teicoplanin and telavancin.

We made all of our potency determinations under standard mycobacterium culture conditions, i.e., Middlebrook 7H9 broth with incubation at 37°C. Because the CLSI guidelines suggest the use of cation-adjusted Mueller-Hinton broth and incubation at 30°C for clinical drug susceptibility testing of rapidly growing mycobacteria (21), we also tested our antibiotic combination under additional conditions (see Tables S1 to S4 in the supplemental material). The drugs showed synergy under many of the conditions tested. However, we observed that assay conditions, including media composition, absence of detergent, and incubation temperature, can strongly affect the MICs (Tables S1 to S4).

In conclusion, vancomycin and clarithromycin exhibited a strong synergistic effect *in vitro* against all M. abscessus strains tested, suggesting potential clinical application. Studies in animals and clinical evaluation of efficacy are required to assess the usefulness of this novel combination *in vivo*. Furthermore, we identified effects of assay conditions on antibiotic potency, highlighting a need for further investigations to improve the predictive value of the *in vitro* growth inhibition assay for clinical outcome.

## Supplementary Material

Supplemental material
