# Supplementary material for: Vancomycin and Clarithromycin Show Synergy against Mycobacterium abscessus In Vitro
Source: Antimicrob Agents Chemother. 2017 Nov 22;61(12):e01298-17. doi: 10.1128/AAC.01298-17 (PMC5700366; doi:10.1128/AAC.01298-17)
Supplement: Supplemental material [file supp_61_12_e01298-17__index.html]

Supplemental material 

# Vancomycin and Clarithromycin Show Synergy against *Mycobacterium abscessus In Vitro*

## Supplemental material

- Supplemental file 1 -

  Supplemental Tables S1 to S4

  PDF, 309K
