# Supplementary material for: Vancomycin and Clarithromycin Show Synergy against Mycobacterium abscessus In Vitro
Source: Antimicrob Agents Chemother. 2017 Nov 22;61(12):e01298-17. doi: 10.1128/AAC.01298-17 (PMC5700366; doi:10.1128/AAC.01298-17)
Supplement: Supplemental material [file AAC.01298-17_zac012176695s1.pdf]

SUPPLEMENTAL MATERIAL

TABLE S1 *In vitro* synergistic effect of clarithromycin (CLR) with vancomycin (VAN) in Middlebrook 7H9 broth at 30°C against two *M. abscessus* type strains with inducible clarithromycin resistance under induced conditions.

**Strains**	**Day**	**MIC (µM)**				**FICI**[a]
		**alone**		**in combination**		
		**CLR**	**VAN**	**CLR**	**VAN**	
**(A) Pre-incubation induction assay**						
M. abscessus subsp. *abscessus* ATCC 19977	3	200	>100	6.25	12.5	<0.16
M. abscessus subsp. *bolletii* CCUG 50184T	3	>200	200	6.25	25	<0.16
**(B) Standard inducible resistance assay**						
M. abscessus subsp. *abscessus* ATCC 19977	3	50	200	6.25	12.5	0.19
	7	>200	>200	25	25	<0.25
	14	>200	>200	50	25	<0.38
M. abscessus subsp. *bolletii* CCUG 50184T	3	100	>200	6.25	50	<0.31
	7	200	>200	6.25	50	<0.28
	14	>200	>200	50	50	<0.5

[a] FICI was calculated as [(MIC of clarithromycin in combination/MIC of clarithromycin alone) + (MIC of vancomycin in combination/MIC of vancomycin alone)]. FICI ≤ 0.5 is considered a synergistic interaction. A less than (<) preceding a FICI score indicates that an MIC of drug alone was higher than the greatest concentration tested, which was used in FICI calculation.

TABLE S2 MIC (μM) of clarithromycin (CLR) and vancomycin (VAN) alone or in combination in Middlebrook 7H9 broth against four *M. abscessus* strains at 37°C and 30°C.

Isolate code	*M. abscessus* subspecies	Day	37°C					30°C				
			alone		in combination		FICI[a]	alone		in combination		FICI[a]
			CLR	VAN	CLR	VAN		CLR	VAN	CLR	VAN	
ATCC 19977	*abscessus*	3	6.25	25	0.39	3.12	0.19	50	200	6.25	12.5	0.19
CCUG 50184T	*bolletii*	3	12.5	12.5	0.78	1.56	0.19	100	>200	6.25	50	<0.31
CCUG 48898T	*massiliense*	3	1.56	50	0.39	3.12	0.31	25	200	6.25	25	0.38
Bamboo	*abscessus*	3	1.56	25	0.20	3.12	0.25	3.12	200	0.39	50	0.38

[a] FICI was calculated as [(MIC of clarithromycin in combination/MIC of clarithromycin alone) + (MIC of vancomycin in combination/MIC of vancomycin alone)]. FICI ≤ 0.5 is considered a synergistic interaction. A less than (<) preceding a FICI score indicates that an MIC of drug alone was higher than the greatest concentration tested, which was used in FICI calculation.

TABLE S3 MIC (μM) of clarithromycin (CLR) and vancomycin (VAN) alone or in combination in Cation-adjusted Mueller-Hinton broth with 0.05% Tween80 against *M. abscessus* subsp. *abscessus* ATCC 19977 at 37°C and 30°C.

Isolate code	*M. abscessus* subspecies	Day	37°C					30°C				
			alone		in combination		FICI[a]	alone		in combination		FICI[a]
			CLR	VAN	CLR	VAN		CLR	VAN	CLR	VAN	
ATCC 19977	*abscessus*	3	3.12	25	0.78	3.12	0.38	6.25	100	1.56	25	0.50
		14	25	50	1.56	3.12	0.13	>200	>200	no synergy[b]		

[a] FICI was calculated as [(MIC of clarithromycin in combination/MIC of clarithromycin alone) + (MIC of vancomycin in combination/MIC of vancomycin alone)]. FICI ≤ 0.5 is considered a synergistic interaction. A less than (<) preceding a FICI score indicates that an MIC of drug alone was higher than the greatest concentration tested, which was used in FICI calculation.

[b] Synergy could not be evaluated as we did not achieve 90% growth inhibition with the concentrations tested.

TABLE S4 MIC (µM) of clarithromycin (CLR) and vancomycin (VAN) alone or in combination in Cation-adjusted Mueller-Hinton broth against *M. abscessus* subsp. *abscessus* ATCC 19977 at 37ºC and 30ºC.

Isolate code	*M. abscessus* subspecies	Day	37ºC					30ºC				
			alone		in combination		FICI[a]	alone		in combination		FICI[a]
			CLR	VAN	CLR	VAN		CLR	VAN	CLR	VAN	
ATCC 19977	*abscessus*	3	12.5	>200	3.12	25	<0.38	50	>200	no synergy[b]		
		14	>200	>200	3.12	50	<0.27	>200	>200	no synergy		

[a] FICI was calculated as [(MIC of clarithromycin in combination/MIC of clarithromycin alone) + (MIC of vancomycin in combination/MIC of vancomycin alone)]. FICI ≤ 0.5 is considered a synergistic interaction. A less than (<) preceding a FICI score indicates that an MIC of drug alone was higher than the greatest concentration tested, which was used in FICI calculation.

[b] The experiments shown above were carried out with 10^7 CFU/ml inoculum (consistent with the inoculum used for the other experiments described in this report). As no synergistic effect was observed at 30ºC, we repeated the experiment with a lower inoculum (10^5 CFU/ml) as suggested by CLSI. At day 3, MIC for clarithromycin alone was 6.25 µM and MIC for vancomycin alone was >200 µM; synergy was not observed with the lower inoculum.
